# Energy-Efficient Route Planning Method for Ships Based on Level Set

**DOI:** 10.3390/s25020381

**Published:** 2025-01-10

**Authors:** Jiejian Zhu, Haiqing Shen, Qiangrong Tang, Zhong Qin, Yalei Yu

**Affiliations:** 1 The School of Shipping and Maritime Studies, Guangzhou Maritime University, Guangzhou 510725, China; 2112216341@e.gzhu.edu.cn (J.Z.); qrtang@gzmtu.edu.cn (Q.T.); 2 The School of Civil Engineering and Transportation, Guangzhou University, Guangzhou 510006, China; qinz@gzhu.edu.cn; 3 The Department of Aeronautical and Automotive Engineering, Loughborough University, Loughborough LE11 3RH, UK; y.yu2@lboro.ac.uk

**Keywords:** ship, level set method, energy saving, path planning

## Abstract

To reduce the fuel consumption of ships’ oceanic voyages, this study incorporates the influence of ocean currents into the traditional level set algorithm and proposes a route planning algorithm capable of identifying energy-efficient routes in complex and variable sea conditions. The approach introduces the influence factor of ocean current to optimize routing in dynamically changing marie environments. First, models for the energy consumption of ships and flow fields are established. The level set curve is then evolved by integrating the flow environment and energy consumption gradient, solving the Hamilton–Jacobi equation with energy consumption parameters. The optimal path is subsequently determined through backtracking along the energy consumption gradient, enabling energy-efficient route planning from the starting point to the endpoint in complex ocean conditions. To verify the effectiveness of the proposed algorithm, its performance is evaluated through two case studies, comparing energy consumption under different environmental conditions. The experimental results demonstrate that, compared to the shortest path method based on the level set algorithm, the proposed approach achieves an energy saving rate of approximately 2.1% in obstacle-free environments and 1.4% in environments with obstacles.

## 1. Introduction

In recent years, the issue of ship energy consumption has garnered significant attention. Most marine vessels rely on fuel as their primary power source, and their operational range is constrained by the amount of fuel they carry [1]. The efficient utilization of limited energy resources during long-distance voyages remains a critical challenge. Notably, some large container ships consume over 150 tons of fuel daily, incurring costs of approximately 78,000 US dollars per day [2,3]. With the advancement of China’s maritime power strategy, reducing ship energy consumption not only contributes to environmental protection, but also plays a vital role in achieving its strategic goals. Concurrently, the International Maritime Organization (IMO) and governments worldwide have introduced new conventions and standards aimed at enhancing energy efficiency and reducing emissions in shipping. To address this issue, scholars have proposed various methods for reducing the energy consumption of marine vessels. Among these, route planning has emerged as a particularly effective approach. This method not only mitigates the impact of complex oceanic conditions on ship energy consumption but also enhances navigational safety, thereby contributing to the sustainable development of China’s maritime industry.

Ship route planning aims to identify an optimal or suboptimal route that connects a starting point to an endpoint while avoiding static obstacles in the environment. Given a ship’s navigation environment, route planning algorithms are essential for preventing collisions and achieving goals such as time optimization [4,5] and energy consumption minimization. Among these objectives, energy efficiency is particularly significant, especially on long-distance ocean voyages where the issue of energy consumption is a critical concern. To address the challenges of ship route planning, scholars have proposed various algorithms for optimal route planning. The artificial potential field (APF) method, for instance, simulates object movement by constructing virtual attractive and repulsive forces. Attractive forces guide the ship toward the target, while repulsive forces prevent collisions with obstacles, thus identifying the optimal path [6]. Petres [7] extended this method by integrating a ship dynamic model to test collision avoidance under varying wind conditions, successfully generating the shortest distance path. However, the APF method is highly dependent on the accuracy of environmental data, and incomplete or erroneous information can lead to deviations in route planning. Liu [8] introduced the use of Self-Organizing Maps (SOM) to model the environment, constructing an adaptive repulsion field that updates environmental information dynamically during navigation. This method achieves real-time obstacle avoidance but faces limitations in complex scenarios such as oscillating obstacles or narrow passages, where path instability or the inability to identify a feasible route may occur. The genetic algorithm (GA) is another prominent approach, inspired by the process of biological evolution. GA encodes potential solutions and evaluates their ability to identify the optimal route. Kuhlemann [9] incorporated the effects of wind and waves into a GA-based ship routing model, aiming to minimize costs while addressing weather conditions, restricted areas, and arrival time constraints. This approach also adjusts ship speed to minimize fuel consumption while ensuring navigational safety. However, GA can suffer from issues such as premature convergence and local optima, limiting its effectiveness in practical applications. The ant colony optimization (ACO) algorithm, which mimics the foraging behavior of ants, has also been applied in ship route planning. Lazarowska [10,11] developed an ACO-based model that avoids obstacles such as land, shoals, and buoys, implementing collision avoidance strategies to identify the optimal path. However, ACO is hindered by slow convergence rates and a tendency to settle in local optima, particularly in complex environments. Energy-efficient route planning has also been extensively studied. Huynh and Kularatne [12,13] employed A*-based algorithms to plan energy-efficient routes, but these algorithms face challenges in large-scale or complex maps due to the generation of excessive unnecessary nodes, which increase computational costs and processing time. Ding et al. [14] proposed an energy-efficient route planning and tracking control method based on the particle swarm optimization (PSO) algorithm, addressing navigational safety and energy consumption in complex ocean environments. Their approach establishes mathematical models for unmanned surface vehicle (USV) motion and environmental factors, but its accuracy is limited in dynamically changing marine conditions, where real-time environmental changes are not effectively captured. This can lead to discrepancies between predicted and actual energy consumption. Wang [15] proposed an improved artificial potential field algorithm that integrates vessel speed to plan energy-saving paths under the influence of wind and currents. While effective, the APF algorithm remains prone to the “local minima” problem, where obstacles can lead to planning failures. Dong [16] introduced a new double ant colony algorithm (NDACA) with a dynamic feedback mechanism to enhance pheromone updates and incorporate energy consumption models for improved efficiency. However, this method has high computational complexity and reduced adaptability as environmental complexity increases. Pan [17] proposed a hybrid genetic–ant colony algorithm that combines the strengths of GA and ACO to enhance global search capabilities. By dynamically adjusting iteration thresholds, this approach accelerates population evolution and considers the influence of flow fields on energy consumption. However, the algorithm’s complexity and computation time can limit its practicality for energy consumption optimization in large-scale or rapidly changing marine environments. In such cases, the algorithm struggles to adjust paths in real time, hindering its ability to consistently optimize energy efficiency. Shu et al. [18] considered the large proportion of ships entering and leaving estuarine ports worldwide. Using ocean currents and tides to enter and leave estuarine ports can save energy and reduce fuel consumption and pollution emissions, but there is no better planning of shipping schedules and use of tides in and out of ports to improve the energy efficiency of ships.

The level set algorithm is a numerical technique designed to address interface evolution problems. Initially proposed by Osher and Sethian in 1988, it has been widely applied in computer vision, image processing, and route planning. The core concept of the level set method is to represent a curve as an implicit function, transforming the problem of curve evolution into the resolution of partial differential equations (PDEs). By leveraging extensive knowledge and experience in numerical simulation and computational fluid dynamics, the level set method effectively addresses these PDEs [19,20,21,22]. In the context of route planning, the level set method is a powerful tool for solving optimal path problems. The objective of route planning is to identify an optimal route that avoids obstacles and connects a starting point to an endpoint within a given environment. The level set algorithm achieves this by converting the route planning problem into a curve evolution problem. Specifically, the free space in the planning domain is represented as an implicit function, while the starting and ending points are defined as initial and goal conditions. By controlling the curve’s evolution rate—based on objectives such as minimizing distance, energy, or avoiding obstacles—an optimal path is generated from the starting to the ending point. Rhoads [23] approached the extreme curve field by indirectly solving the Hamilton–Jacobi–Bellman equation, using the feedback control rate derived from the Euler–Lagrange equation (with boundary points). This method successfully identified the time-optimal path for robotic systems. Similarly, Tsitsiklis et al. [24] converted the route planning problem into a discrete Hamilton–Jacobi PDE, employing the first-order fast marching algorithm to solve it. The optimal path was then extracted using a gradient descent method. This approach effectively handles complex environments, offering strong convergence, stability, and the ability to find globally optimal solutions. Petres et al. [25,26,27] proposed a continuous route planning framework using the fast-marching algorithm. This method incorporates an anisotropic cost function, defining an outward force field as a constraint within the optimization problem. It not only accounts for obstacle avoidance, but also accommodates dynamically changing environmental factors. In the maritime domain, the level set method has been employed for ship route planning. For example, Lolla et al. [28] addressed the route planning problem for underwater vehicles navigating strong ocean currents. By moving beyond grid-based search methods, this approach ensured theoretical feasibility in high-current environments. Zeng et al. [29] introduced ship maneuverability constraints into the level set framework. This method integrated constraints such as steering capabilities, speed limits, and navigational safety into the level set equation, producing numerically stable and feasible routes that satisfy dynamic requirements. Ma Yong et al. [30] utilized a second-order alternating evolution approach to solve the Hamilton–Jacobi equation with time parameters. They developed a second-order polygonal optimization reachable boundary using Newton’s mean difference interpolation method. By leveraging reachable boundary information and the path backtracking equation, they successfully planned time-optimal paths in dynamic environments. Xiao [31] proposed a time-optimal route planning algorithm based on reachability theory, considering both moving and deformable obstacles. By combining reachability theory with the level set method for numerical solutions, this approach achieved time-optimal paths while ensuring collision avoidance.

Although the level set method has been successfully applied to route planning problems, current research remains limited. Most studies focus on optimizing the shortest path or minimizing travel time, with relatively few focusing on route planning from the perspective of energy consumption. To address this gap, this paper proposes an improved level set algorithm that incorporates the influence of ocean currents. By combining the flow environment [32] and energy consumption gradient, the level set curve is evolved to solve the Hamilton–Jacobi equation with energy consumption parameters. The optimal path is then determined based on the direction of the energy consumption gradient, enabling energy-efficient route planning for ships navigating complex ocean environments. The effectiveness of the proposed algorithm is validated by comparing it with the shortest path algorithm based on the traditional level set method. The results demonstrate its capability in achieving energy-saving route planning. The structure of this paper is as follows: Section 2 describes the problem formulation. Section 3 introduces the theoretical foundation of the level set method. The numerical simulations and results are presented in Section 4. Finally, the conclusions are provided in Section 5.

## 2. The Formulation of Energy-Saving Route Planning

### 2.1. Problem Statement

**Figure 1:**
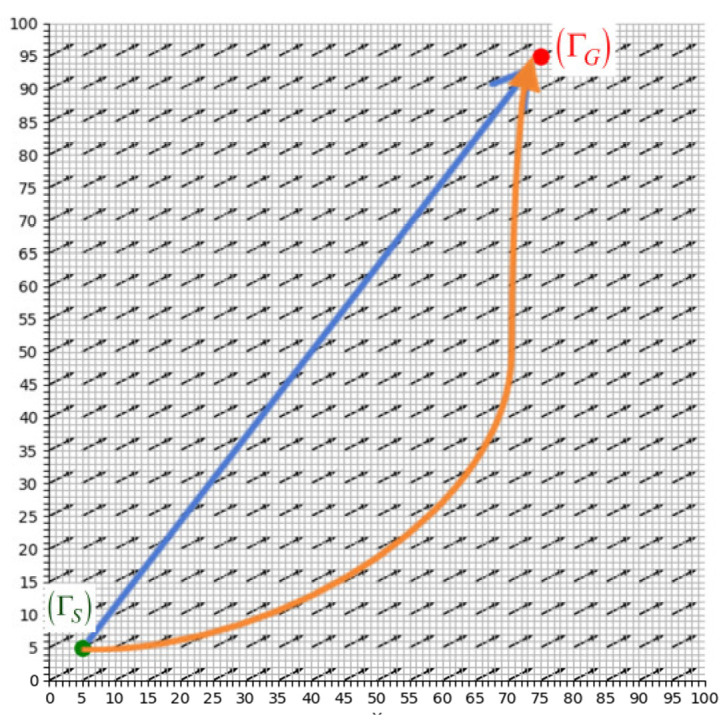
Ship route planning problem.

**Figure 2:**
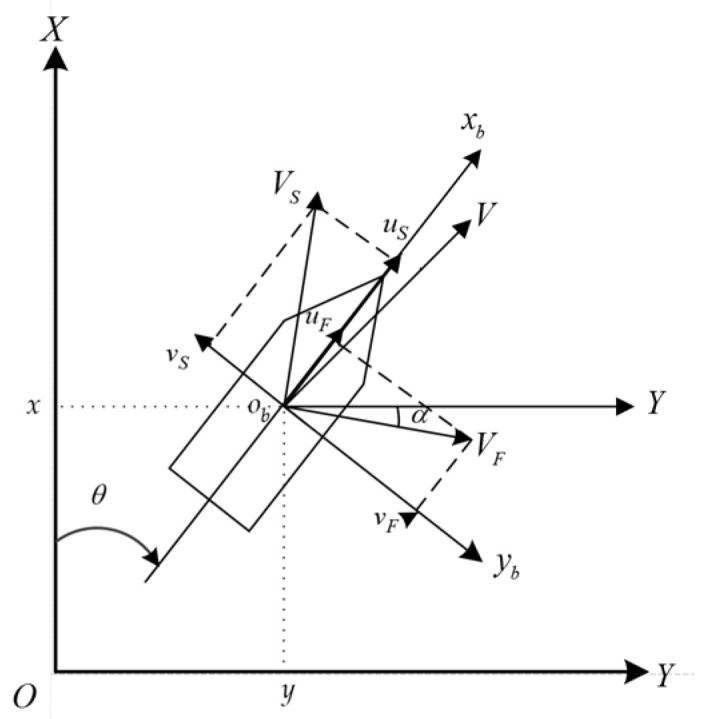
Ship motion coordinate system.

The path planning of a ship with ocean currents can be illustrated in Figure 1. In a two-dimensional space, let Γ represent a position vector, with the starting point being $\left(\Gamma_{S}\right)$ and the target point being $\left(\Gamma_{G}\right)$. In Figure 1, the black arrows represent the flow fields environment, the green solid circles indicate the starting point $\left(\Gamma_{S}\right)$, and the red solid circles indicate the endpoint $\left(\Gamma_{G}\right)$. The arrowed curves represent the safe and reasonable path trend, planned using the flow fields, and the planned path is the blue straight line trend with arrows in the figure. Also, consider the relationship between the ship and the sea flow fields $V_{F}$. As shown in Figure 2, O−XY is the earth coordinate system and $o_{b}-x_{b}y_{b}$ is the ship coordinate system. The navigation speed of the vessel relative to the ocean current, $V_{S}$, can be decomposed into the following components: the speed $u_{S}$ in the $x_{b}$ direction and the speed $v_{S}$ in the $\;y_{b}$ direction. The external sea current $V_{F}$ can be decomposed into $u_{F}$ in the $x_{b}$ direction and $v_{F}$ in the $\;y_{b}$ direction.

The actual navigation speed of the vessel, V, under the influence of $V_{F}$ in the flow fields, is the composite speed of the ship and is obtained by $\vec{V}=\vec{V_{S}}+\vec{V_{F}}$. The ship adjusts its course under the influence of the flow fields to safely navigate from the starting point $\left(\Gamma_{S}\right)$ to the target point $\left(\Gamma_{G}\right)$, thus the orange energy-saving path line shown in Figure 1 can be obtained.

### 2.2. Flow Fields Model

The flow fields model typically describes the spatial and temporal variations in the flow velocity vector. This velocity vector can be decomposed into components within the hull coordinate system, facilitating the analysis of the direct impact of flow on the ship’s movement. The mathematical formulation of the flow fields model is presented in Equations (1)–(5).

(1) $$V_{F_{X}}\left(x,y,t\right)=V_{F}\left(x,y,t\right)\sin\left(\alpha \left(x,y,t\right)\right)$$

(2) $$V_{F_{Y}}\left(x,y,t\right)=V_{F}\left(x,y,t\right)\cos\left(\alpha \left(x,y,t\right)\right)$$

(3) $$u_{F}\left(x,y,t\right)=V_{F_{X}}\left(x,y,t\right)\sin\left(\theta \left(x,y,t\right)\right)-V_{F_{Y}}\left(x,y,t\right)\cos\left(\theta \left(x,y,t\right)\right)$$

(4) $$v_{F}\left(x,y,t\right)=V_{F_{X}}\left(x,y,t\right)\cos\left(\theta \left(x,y,t\right)\right)+V_{F_{Y}}\left(x,y,t\right)\sin\left(\theta \left(x,y,t\right)\right)$$

(5) $$V_{F}\left(x,y,t\right)=\sqrt{u_{F}{\left(x,y,t\right)}^{2}+v_{F}{\left(x,y,t\right)}^{2}}$$

where

$V_{F}\left(x,y,t\right)$: flow speed magnitude, obtained from actual data.

$V_{F_{x}}\left(x,y,t\right)$: component of flow speed in the X direction of the earth coordinate system.

$V_{F_{y}}\left(x,y,t\right)$: component of flow speed in the Y direction of the earth coordinate system.

$u_{F}\left(x,y,t\right)$: component of flow speed in the $x_{b}$ direction of the ship coordinate system.

$v_{F}\left(x,y,t\right)$: component of flow speed in the $y_{b}$ direction of the ship coordinate system.

$\alpha \left(x,y,t\right)$: flow direction angle, i.e., the angle between the flow velocity vector and the earth coordinate system.

$\theta \left(x,y,t\right)$: rotation angle of the ship coordinate system relative to the earth coordinate system.

### 2.3. Energy Consumption Model

Taking into account the influence of the flow fields on ship navigation, a route planning algorithm was developed to optimize energy consumption within the flow fields. To enhance the computational efficiency of the route planning process, the grid method is employed to discretize the navigational environment. During the modeling phase, the number of rows and columns in the grid is determined based on a selected sea area interval and the desired grid resolution. The calculation formula [33] for this process is provided in Equation (6).

(6) $$\left\{\begin{array}{l} Col.Num=\frac{Lon.Max-Lon.Min}{Grid\;accuracy}\\ Row.Num=\frac{Lat.Max-Lat.Min}{Grid\;accuracy} \end{array}\right.$$

Among them, *Lon.Max* and *Lat.Max* are the upper boundaries. Lon.Min and Lat.Min are the lower boundaries. Grid   accuracy is the grid precision. We chose a grid precision of 0.5 nautical miles × 0.5 nautical miles to build the model.

During navigation, the water-facing area of the vessel changes based on its heading. This water-facing area can be decomposed into directional components, as expressed in Equations (7) and (8).

(7) $$S_{X}=d\times \left(L\cos\theta +B\sin\theta \right)$$

(8) $$S_{Y}=d\times \left(L\sin\theta +B\cos\theta \right)$$

where L,B,d represent the length, width and draft of the ship, respectively. θ denotes the angle between the direction of motion and the X-axis. $S_{X}$ is the projected area in the X direction and $S_{Y}$ is the projected area in the Y direction.

The energy consumption depends on the area of the front in the X,Y directions. The energy consumption formula is shown in Equations (9)–(11).

(9) $$E_{X}=\frac{1}{2}\rho C_{f}S_{X}\left(V\sin\theta -V_{F_{X}}\right)T_{s}$$

(10) $$E_{Y}=\frac{1}{2}\rho C_{f}S_{Y}\left(V\cos\theta -V_{F_{Y}}\right)T_{s}$$

(11) $$E=E_{X}+E_{Y}$$

where $C_{f}$ represents the seawater drag coefficient, $V_{F_{X}}$ denotes the flow fields component in X, $V_{F_{Y}}$ is the flow fields component in Y, and V denotes the combined speed of the ship. $E_{X}$ represents the energy consumed by the ship in the X direction, and $E_{Y}$ is the energy consumed by the ship in the Y direction. E is the total energy consumed by the ship during its voyage. $T_{S}$ is the time that the ship travels during its course.

When the ship sails from $S_{0}$ to $S_{1}$, the energy consumption is $E\left(S_{1},\;S_{0}\right)$, and the required energy is as follows:

(12) $$E\left(S_{1},S_{0}\right)=\int_{0}^{T_{s}}\left(E_{X}+E_{Y}\right)dt$$

(13) $$E=\overset{n}{\underset{i=1}{\sum }}E_{i,i+1}$$

## 3. Path Planning Theory Based on Level Set

### 3.1. Level Set Theory

**Figure 3:**
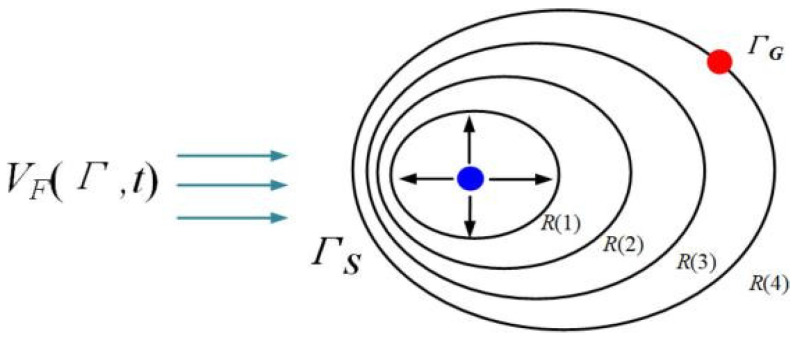
The reachable area and boundary of the ship.

Let $V_{F}\left(\Gamma ,t\right)$ be the flow fields at time t. As shown in Figure 3, $R\left(1\right)$ represents the boundary reached after the first time unit, which is the farthest point the ship can reach in any direction from its starting point $\left(\Gamma_{S}\right)$. Similarly, at each point on the reachable boundary $R\left(1\right)$, the ship can choose to sail in any direction.

After one unit of time, each point on the reachable boundary $R\left(1\right)$ has its own reachable region. The union of these regions forms the ship’s reachable area within two units of time from $\Gamma_{S}$, and the corresponding reachable boundary is $R\left(2\right)$ in Figure 3. This process repeats iteratively until the reachable boundary first intersects the target point $\Gamma_{G}$.

The point at which the level set function $\varphi \left(\Gamma \left(t\right),t\right)$ evolves over time, and where its value is zero, can be expressed as $\varphi \left(\Gamma \left(t\right),t\right)=0$, indicating the movement of the boundary over time *t*. At any point $\Gamma \left(t\right)=\left(x\left(t\right),y\left(t\right)\right)$ on the reachable boundary $R\left(t\right)$, the ship is influenced by its own driving speed $V_{U}(\Gamma ,t)$, which can be directed in any direction. Assume an external field function $V_{F}(\Gamma ,t)$, indicating that every point on the curve in 2D space is affected by that velocity component. Thus, under the combined influence of $V_{U}(\Gamma ,t)$ and $V_{F}(\Gamma ,t)$, the points on the curve evolve with time. Therefore, the motion control equation of the ship is shown in Equation (14) below:

(14) $$\frac{d\Gamma \left(t\right)}{dt}=V_{S}\left(\Gamma ,t\right)+V_{F}\left(\Gamma ,t\right)$$

taking the derivative of $\varphi \left(\Gamma \left(t\right),t\right)=0$, and then we obtain Equation (15).

(15) $$\frac{\partial \varphi \left(\Gamma ,t\right)}{\partial t}+\left[V_{S}\left(\Gamma ,t\right)\vec{N_{t}}+V_{F}\left(\Gamma ,t\right)\right]\nabla \varphi_{t}\left(\Gamma ,t\right)=0$$

where $\nabla \varphi_{t}$ represents the gradient of $\varphi \left(\Gamma ,t\right)$, and the unit normal vector of the zero level set curve R(t) in Equation (15) is $N_{t}=\nabla \varphi_{t}/|\nabla \varphi |$, this vector also defines the direction in which the ship is moving with velocity V. Initial condition $\varphi (\Gamma ,0)=\left|\Gamma -\Gamma s\right|,\Gamma s$ represents the starting point. Equation (15) is known as the Hamilton–Jacobi equation [34].

Choose $H\left(x,y,\nabla \varphi \right)=\left[V_{S}\left(\Gamma ,t\right)\vec{N_{t}}+V_{F}\left(\Gamma ,t\right)\right]\nabla \varphi_{t}\left(\Gamma ,t\right)$ then $H\left(x,y,\nabla \varphi \right)=V\left(x,y\right)*\;\|\nabla \varphi \|$ can be simplified to $\partial \varphi /\partial t+H\left(x,y,\nabla \varphi \right)=0$, where H is the Hamilton function, reflecting the influence of the environment and constraints on the path.

To discretize the above equation, the computational domain is divided into grids, with the grid nodes represented by $\left(x_{i,}y_{j}\right)$, and the time-derivative term is discretized. The specific formula is as shown in Equations (16) and (17) below:

(16) $$\frac{\varphi_{ij}^{n+1}-\varphi_{ij}^{n}}{\Delta t}+H\left(x_{i},y_{j},\nabla \varphi_{ij}^{n}\right)=0$$

(17) $$H\left(x_{i},y_{j},\nabla \varphi_{ij}^{n}\right)=V\left(x_{i},y_{j}\right)\times \left|\nabla \varphi_{ij}^{n}\right|$$

Given the initial condition, the solution is updated iteratively until φ converges or the maximum number of iterations is reached. The optimal energy-saving path is then obtained by calculating the gradient of the numerical solution of φ and backtracking along the gradient line starting from the endpoint.

### 3.2. The Energy Consumption Gradient Is Used to Solve the Energy-Saving Path

The energy consumption gradient ∇E is a vector field that indicates the direction of the most rapid change in energy consumption within a given spatial region. In the context of route planning, the energy consumption gradient serves as a guide to identify the path with the slowest increase in energy consumption, which corresponds to the optimal path. Specifically, the energy consumption gradient reflects both the magnitude and direction of energy consumption change per unit distance. In level set theory, the optimal energy-saving path is obtained by calculating the energy consumption gradient and backtracking along the gradient line starting from the endpoint.

For the energy consumption function $E\left(x_{,}y\right)$, where E is the energy consumption at position $\left(x_{,}y\right)$, the five-point difference formula is used to calculate the energy consumption gradient ∇E in the X and Y directions:

(18) $$\frac{\partial \nabla E}{\partial x}\approx \frac{E\left(x+2\Delta x,y\right)-8E\left(x+\Delta x,y\right)+8E\left(x-\Delta x,y\right)-E\left(x-2\Delta x,y\right)}{12\Delta x}$$

(19) $$\frac{\partial \nabla E}{\partial y}\approx \frac{E\left(x,y+2\Delta y\right)-8E\left(x,y+\Delta y\right)+8E\left(x,y-\Delta y\right)-E\left(x,y-2\Delta y\right)}{12\Delta y}$$

where Δx and Δy in Equations (18) and (19) represent the mesh step size in the x and y directions, respectively.

At the boundary, the five-point difference formula cannot be applied. Instead, the central difference method is used to calculate the energy consumption gradient.

Formula (20) is used to calculate x-direction boundary points.

(20) $$\frac{\partial \nabla E}{\partial x}\approx \frac{E\left(x+\Delta x,y\right)-E\left(x-\Delta x,y\right)}{2\Delta x}$$

Formula (21) is used to calculate y-direction boundary points.

(21) $$\frac{\partial \nabla E}{\partial y}\approx \frac{E\left(x,y+\Delta y\right)-E\left(x,y-\Delta y\right)}{2\Delta y}$$

Therefore, by integrating the gradient in the X and Y directions, the energy consumption gradient vector can be obtained using Equations (20) and (21).

(22) $$\nabla E=\left(\frac{\partial \nabla E}{\partial x},\frac{\partial \nabla E}{\partial y}\right)$$

The gradient vector indicates the direction of the fastest increase in energy consumption, corresponding to the steepest rise in energy consumption. However, in route planning, the objective is typically to identify the path with the steepest reduction in energy consumption. Therefore, the reverse direction of the gradient should be considered. Ultimately, the optimal energy-saving path can be determined by calculating the gradient of the numerical solution of φ and backtracking along the gradient line from the endpoint.

## 4. Numerical Simulation

Building upon the traditional level set algorithm [35], this paper employs the flow field model to calculate the velocity and direction of the flow fields. By integrating the flow fields environment with the energy consumption gradient, the level set curve is evolved to develop an energy-saving path algorithm based on the level set method. To validate the effectiveness of the proposed algorithm, this section compares the energy consumption differences between the energy-saving path algorithm and the shortest path algorithm in two case studies. The energy-saving performance of the proposed approach is evaluated in varying environmental conditions. First, a comparison of energy consumption between shortest route planning and energy-saving route planning was conducted using real flow fields data in an obstacle-free environment. Subsequently, the energy consumption of both route planning methods was analyzed in a complex environment with static obstacles. This second comparison further verifies the applicability and energy-saving capabilities of the proposed algorithm under complex conditions.

**Figure 4:**
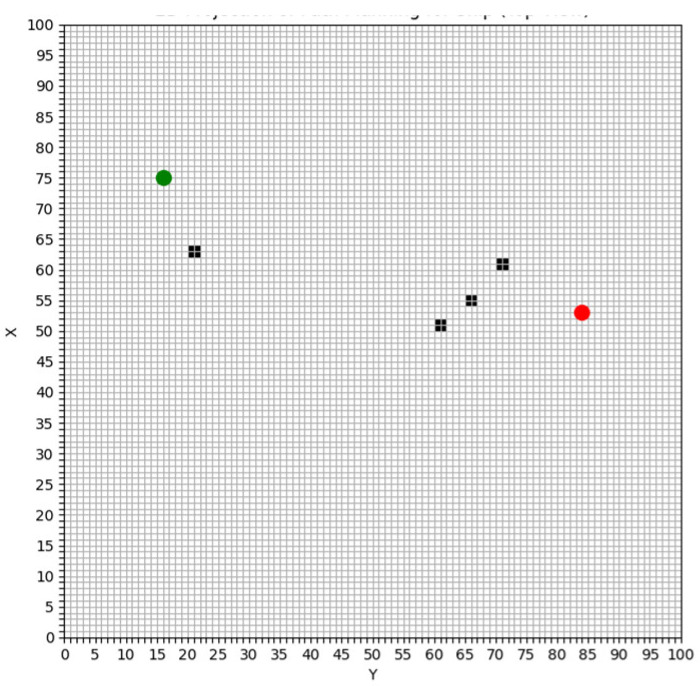
The study area is gridded.

The study area of this paper spans 50 nautical miles from north to south and 50 nautical miles from east to west, divided into a grid of 100 × 100. In the ship simulation experiment, the ship has a length of 185 m, a width of 32.26 m, and a draught of 12 m. The starting position of the ship is set at coordinates (16, 74), and the target position is set at (84, 56). The ship’s speed relative to the water is set at 10 knots, with a seawater density of 1024 kg/m^3^, and a seawater drag coefficient ($C_{f}$) of 15.72. Figure 4 illustrates the gridded study area. The green point represents the starting position, the red point denotes the target position, and the black squares indicate obstacles.

**Figure 5:**
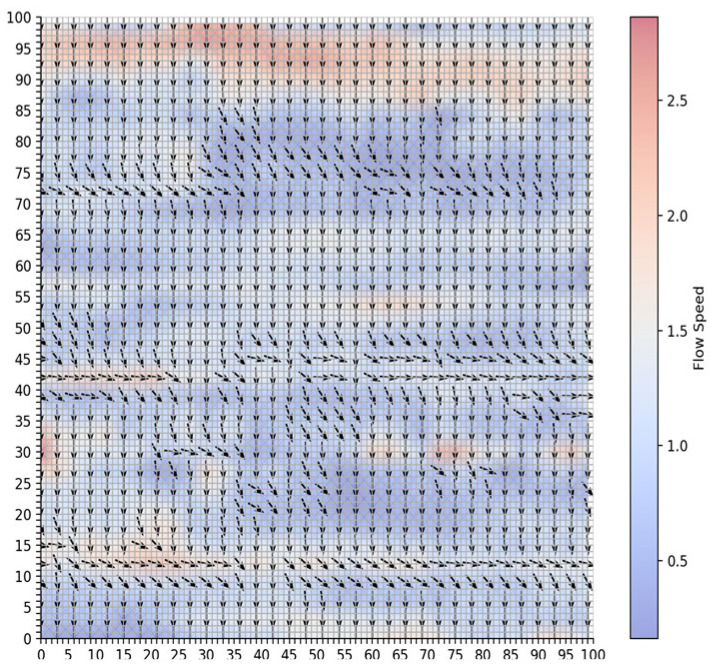
Flow fields environment.

To simulate route planning, this study selected flow fields data from a specific area as the study region to visualize the flow fields, as shown in Figure 5. In the figure, arrows indicate the direction of the flow, while the color represents the flow velocity in knots (kn). The flow velocity magnitude ranges from 0 to 3 *kn*.

### 4.1. Example 1

**Figure 6:**
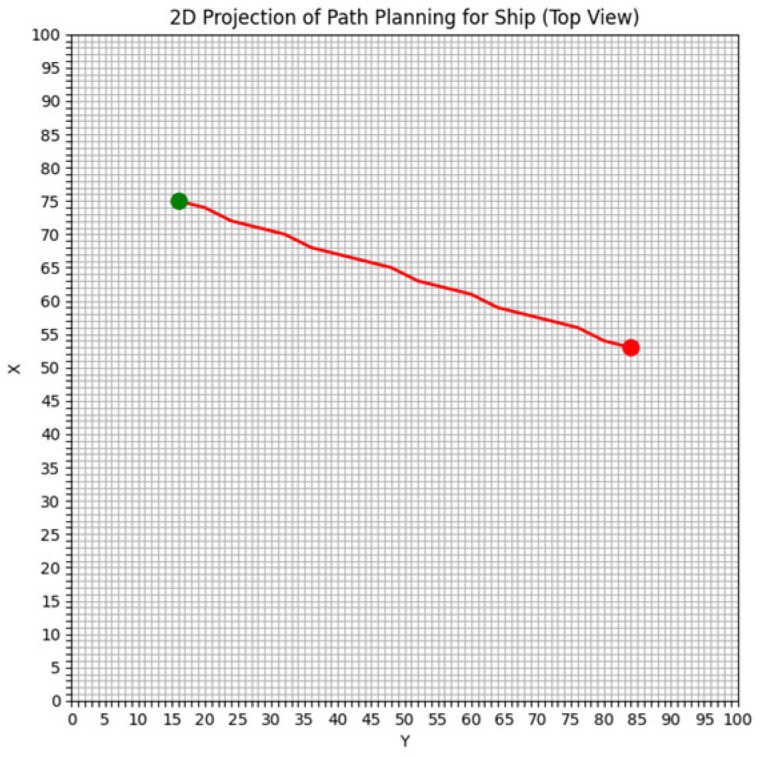
The path of the shortest path algorithm.

**Figure 7:**
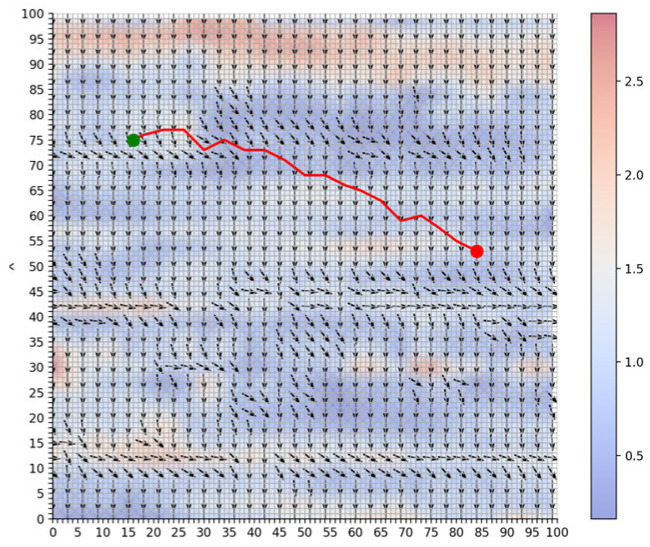
The path of the level set algorithm based on energy saving.

In the numerical simulation one, to verify the effectiveness of the proposed algorithm, we compare the energy consumption differences between the shortest route planning algorithm based on the level set method and the energy-saving path algorithm in an obstacle-free environment. The planned paths are illustrated in Figure 6 and Figure 7.

Three key indicators—route planning distance, total energy consumption, and total time consumption—are used as evaluation criteria to assess the performance of the two algorithms. Based on the route planning results shown in Figure 6 and Figure 7, the three performance indicators for both algorithms are summarized in Table 1.

Both route planning methods used the same starting and ending points during the simulation. Based on the specified environment and energy consumption formula, the energy consumption values for the traditional level set algorithm and the optimized level set algorithm were calculated. Additionally, the energy-saving rate of the energy-saving path algorithm, compared to the shortest path algorithm, was determined. The energy-saving rate is calculated using Equation (23), as provided below:

(23) $$rate=\frac{ACO-EACO}{ACO}\times 100\%$$

where *EACO* represents the ocean current energy consumption of the optimized algorithm, *ACO* denotes the ocean current energy consumption of the shortest path algorithm, and *rate* refers to the energy-saving rate. The shortest path algorithm is guided solely by path length, whereas the optimized level set algorithm, which considers the influence of the flow fields, is guided by energy cost. A path with low ocean current energy consumption does not necessarily correspond to a short path length, and similarly, a shorter path length does not guarantee low energy consumption. For instance, if the shortest path traverses a region of high energy consumption, the overall energy cost will increase. Avoiding such regions, even at the expense of increasing the path length, can significantly reduce energy consumption and may ultimately prove more economical. The shortest path algorithm does not account for flow influences and tends to select regions with higher flow resistance relative to the navigation direction in pursuit of minimal path length. In contrast, the optimized algorithm, based on the traditional level set method, better utilizes favorable downflow directions. Although this approach may slightly increase the path length, it results in lower overall energy consumption. The numerical simulation one results demonstrate that the optimized level set algorithm has a longer sailing time but achieves an energy-saving rate of approximately 2.1%.

### 4.2. Example 2

**Figure 8:**
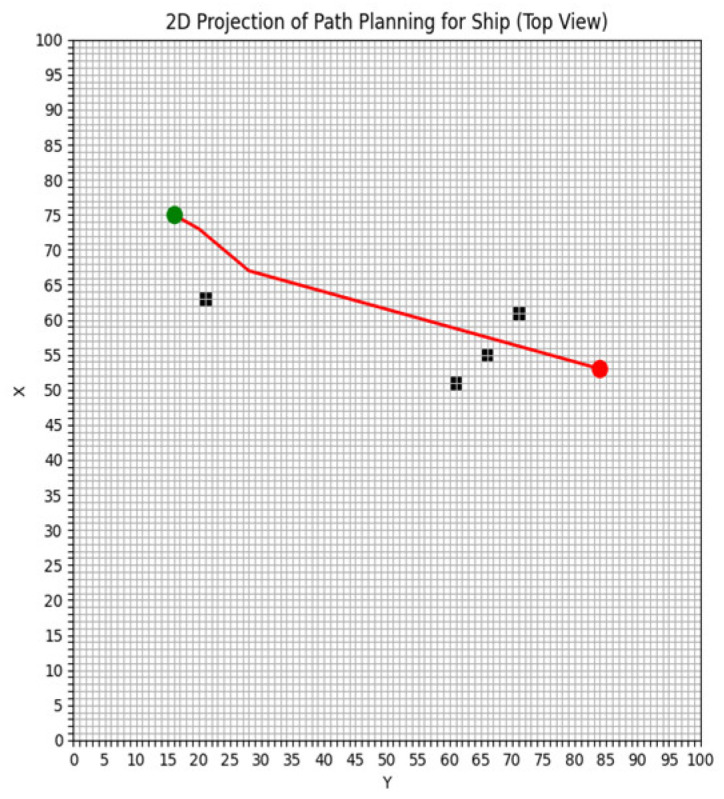
The path of the shortest path algorithm based on energy saving.

**Figure 9:**
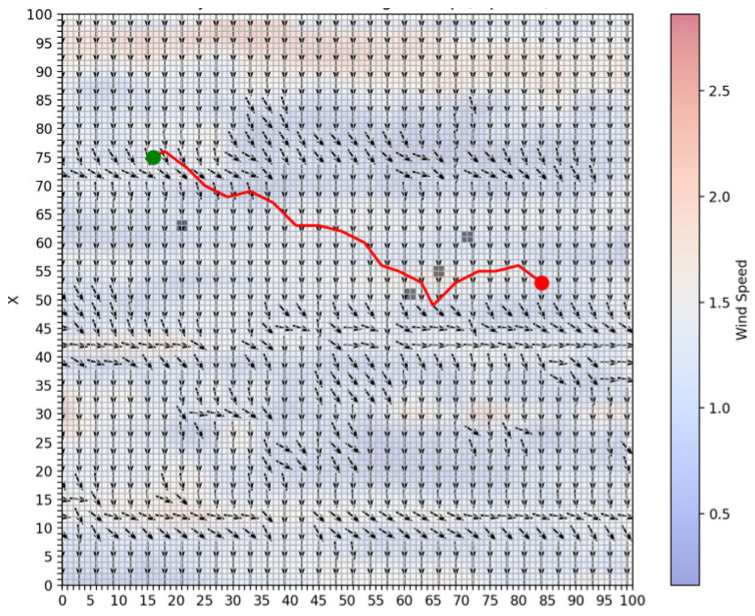
The path of the level set algorithm based on energy saving.

The following numerical simulation compares the energy consumption differences between the energy-saving path algorithm and the shortest path algorithm in the presence of static obstacles. In the numerical simulation two, we calculate the energy consumption difference between the shortest route planning algorithm and the energy-saving route planning algorithm under identical conditions: the same starting and ending positions, departure time, and obstacles. Generally, a ship cannot cross obstacles, and the speed of the ship relative to the water within the obstacle area is zero, and the speed of the flow fields is also zero. During initialization, the velocity of both the ship and the flow fields in the obstacle areas is set to 0. The paths generated by the numerical simulation two are shown in Figure 8 and Figure 9. As in the numerical simulation one, three key indicators—route planning distance, total energy consumption, and total time—are used to evaluate and compare the performance of the two algorithms. Based on the route planning results shown in Figure 8 and Figure 9, the three performance indicators for both algorithms are summarized in Table 2.

As shown in Figure 9, the optimized level set algorithm effectively reduces overall energy consumption despite the “detour” phenomenon, by considering the influence of the flow fields direction and selecting areas with lower navigation resistance. Unlike the shortest path algorithm, the optimized algorithm prioritizes the alignment between the flow fields and the navigation direction, thereby avoiding high-resistance areas and minimizing unnecessary energy consumption. Although the path is slightly longer, the overall energy consumption is significantly lower. The numerical simulation results indicate that the proposed algorithm achieves an energy-saving rate of approximately 1.4%.

## 5. Conclusions

In this paper, a level set-based approach for the energy-efficient route planning of ships is proposed. First, a ship’s energy consumption model and a flow fields model are established. The level set curve is then evolved by incorporating the flow fields environment and energy consumption gradient, solving the Hamilton–Jacobi equation with energy consumption parameters. Finally, the optimal path is identified through path backtracking along the gradient direction of energy consumption, enabling energy-efficient route planning for ships sailing in complex ocean environments. The energy-saving effectiveness of the proposed method was validated through simulations in various environments. Compared to the traditional level set method, the proposed energy-efficient route planning approach demonstrated significant energy saving in both obstacle-free and obstacle-laden environments. In the obstacle-free environment, an energy-saving rate of 2.1% was achieved, while in the environment with obstacles, the energy-saving rate was 1.4%, highlighting its practical significance. Future work will focus on testing the algorithm’s performance in more complex ocean environments, such as those with dynamically changing wind fields.

## Figures and Tables

**Table 1:** Comparison of the parameters of the path without obstacles.

Algorithm Type	Total Distance (n mile)	Total Time (h)	Total Energy Consumption (J)
Shortest Path Algorithm	35.9	2.761	28,436,001,130
Energy-efficient Path Algorithm	39.8	3.069	28,085,241,160

**Table 2:** Comparison of path parameters in the presence of obstacles.

Algorithm Type	Total Distance (n mile)	Total Time (h)	Total Energy Consumption (J)
Shortest Path Algorithm	36.1	2.983	28,736,203,650
Energy-efficient Path Algorithm	43.8	3.658	28,380,363,330

## Data Availability

The data are contained within the article.
